# Supplementary figures and images for: Hyperbaric Oxygen Therapy Reduces Oxidative Stress and Inflammation, and Increases Growth Factors Favouring the Healing Process of Diabetic Wounds
Source: Int J Mol Sci. 2023 Apr 11;24(8):7040. doi: 10.3390/ijms24087040 (PMC10139175; doi:10.3390/ijms24087040)

**Figure S1. Representative images of western blotting bands.**

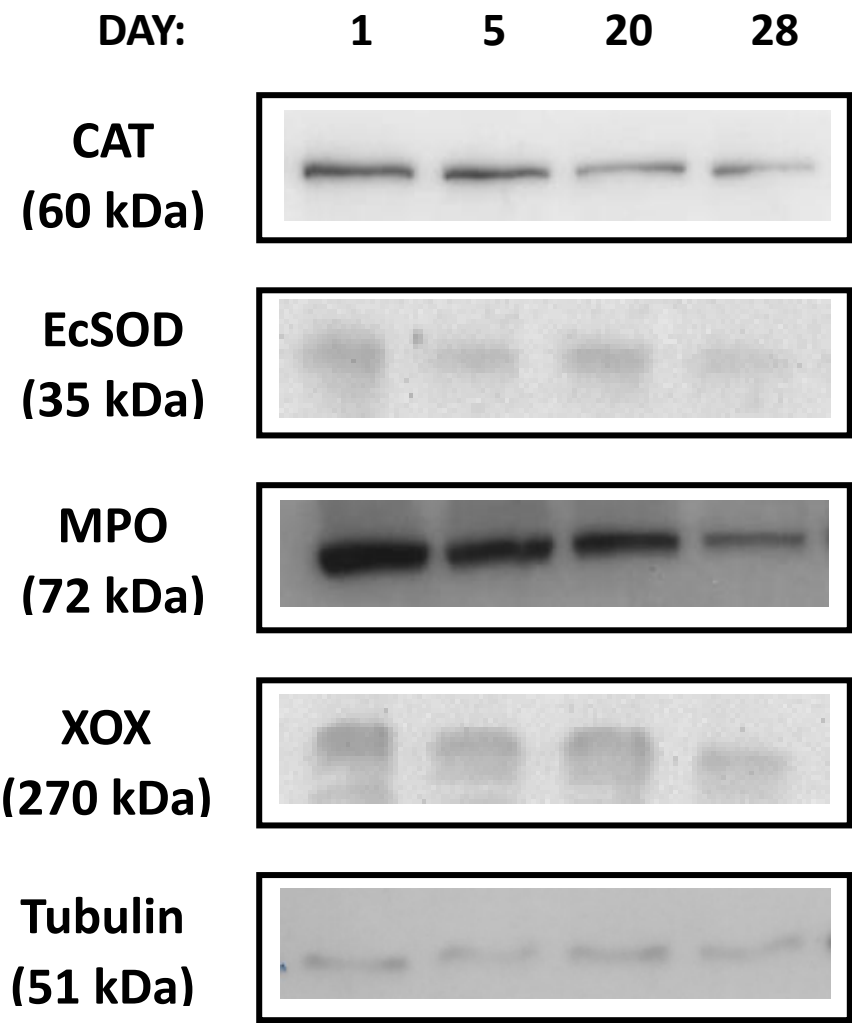

Supplement: Supplementary file 1 [file ijms-24-07040-s001.zip › ijms-2324366-supplementary.pdf]
